# HIV Testing Trends at Visits to Physician Offices, Community Health Centers, and Emergency Departments — United States, 2009–2017

**DOI:** 10.15585/mmwr.mm6925a2

**Published:** 2020-06-26

**Authors:** Karen W. Hoover, Ya-Lin A. Huang, Mary L. Tanner, Weiming Zhu, Naomie W. Gathua, Marc A. Pitasi, Elizabeth A. DiNenno, Suma Nair, Kevin P. Delaney

**Affiliations:** ^1^Division of HIV/AIDS Prevention, National Center for HIV/AIDS, Viral Hepatitis, STD, and TB Prevention, CDC; ^2^Bureau of Primary Health Care, Health Resources & Services Administration, Rockville, Maryland.

In 2019, the U.S. Department of Health and Human Services launched the Ending the HIV Epidemic: A Plan for America (EHE) initiative to end the U.S. human immunodeficiency virus (HIV) epidemic by 2030. A critical component of the EHE initiative involves early diagnosis of HIV infection, along with prevention of new transmissions, treatment of infections, and response to HIV outbreaks (*1*). HIV testing is the first step in identifying persons with HIV infection who need to be engaged in treatment and care as well as persons with a negative HIV test result and who are at high risk for infection and can benefit from HIV preexposure prophylaxis (PrEP) and other prevention services. These opportunities are often missed for persons receiving clinical services in ambulatory care settings (*2*). Data from the 2009–2016 National Ambulatory Medical Care Survey (NAMCS) and 2009–2017 National Hospital Ambulatory Medical Care Survey (NHAMCS) were analyzed to estimate trends in HIV testing at visits by males and nonpregnant females to physician offices, community health centers (CHCs), and emergency departments (EDs) in the United States. HIV tests were performed at 0.63% of 516 million visits to physician offices, 2.65% of 37 million visits to CHCs, and 0.55% of 87 million visits to EDs. The percentage of visits with an HIV test did not increase at visits to physician offices during 2009–2016, increased at visits to CHC physicians during 2009–2014, and increased slightly at visits to EDs during 2009–2017. All adolescents and adults should have at least one HIV test in their lifetime (*3*). Strategies that reduce clinical barriers to HIV testing (e.g., clinical decision supports that use information in electronic health records [EHRs] to order an HIV test for persons who require one or standing orders for routine opt-out testing) are needed to increase HIV testing at ambulatory care visits.

The EHE initiative includes targets of diagnosing ≥95% of HIV infections and prescribing PrEP for ≥50% of persons with indications for PrEP by 2025 (*4*). During 2018, approximately 86% of persons with HIV infection were aware of their infection status, and an estimated 18% of persons with an indication for PrEP were prescribed PrEP (*4*). Routine opt-out HIV testing has been recommended by CDC since 2006 (*3*) and by the U.S. Preventive Services Task Force (USPSTF) as an A-graded preventive service since 2013, with the most recent update in 2019.* Since early 2014, a provision of the Patient Protection and Affordable Care Act has required that third-party health care payers cover HIV testing without a patient deductible or copayment because of the USPSTF A grade.^†^

The most recent data available from NAMCS and NHAMCS were analyzed to estimate the mean annual number of visits by males and nonpregnant females aged 13–64 years to physician offices, CHCs, and EDs, and the percentage of visits at which an HIV test was performed. NAMCS was based on a sample of visits to office-based physicians during 2009–2011 and 2016 and a state-based sampling design during 2012–2015.^§^ NAMCS included a separate sample of visits to CHCs that used a grantee-based sampling design during 2009–2011 and a delivery site design during 2012–2014. NHAMCS was based on a sample of visits to EDs. NAMCS used a three-stage probability design with samples drawn from primary sampling units (PSUs) (geographically defined areas), physician practices or CHCs within PSUs, and patient visits within practices. NHAMCS used a four-stage probability design with samples of PSUs, hospitals within PSUs, clinics within outpatient departments, and patient visits within clinics and emergency service areas. In NAMCS and NHAMCS, medical records from sampled visits were abstracted using a patient record form with checkboxes for important clinical services that were ordered or provided and for the type of visit, including HIV testing, other laboratory testing that required venipuncture, preventive care visits, nonurgent care visits, and diagnoses including HIV infection and pregnancy. Visits for persons with previously diagnosed HIV infection and pregnant women, who are routinely tested for HIV at least once during their pregnancy, were excluded from the analysis. The survey findings were weighted using estimation procedures that resulted in nationally representative estimates of clinical services provided at visits.^¶^ Estimates were stratified by patient demographic and visit characteristics, and 95% confidence intervals were calculated. The percentage of visits with an HIV test was estimated by year for physician offices for 2009–2016, physicians in CHCs for 2009–2014, and EDs for 2009–2017. The percentage of visits with an HIV test was also estimated for persons with private insurance and Medicaid for 2009–2012, 2013–2014, and 2015–2016 for physician offices and EDs and 2009–2012 and 2013–2014 for CHCs; multiple years were combined to increase the statistical reliability of estimates. The categories for the type of payer were based on a hierarchy of private insurance, Medicaid, and other payer types. The statistical significance of temporal trends in HIV testing were assessed by using Cochran-Mantel-Haenszel tests. The statistical significance of differences in HIV testing between subgroups was assessed using Chi-squared tests. All analyses were performed by using SAS-callable SUDAAN (version 11.0.3; RTI International).

During the study periods, males and nonpregnant females made a mean annual 516 million visits to physician offices, 37 million visits to CHCs, and 87 million visits to EDs, with HIV testing performed at 0.63%, 2.65%, and 0.55% of those visits, respectively (Table). HIV testing rates were higher at visits made by persons aged 20–29 years to physician offices and to CHCs compared with visits made by younger or older persons. HIV testing was performed at a larger percentage of visits by non-Hispanic black/African American (black) and Hispanic/Latino (Hispanic) persons than at those by non-Hispanic white (white) persons in physician offices, CHCs, and EDs. HIV testing rates were higher at visits to physician offices, CHCs, and EDs located in metropolitan statistical areas (more urban areas), compared with those located in nonmetropolitan statistical areas (less urban areas). The percentage of visits with an HIV test performed did not increase in physician offices during 2009–2016 (p = 0.0534), increased markedly in CHCs from 0.76% in 2009 to 2.41% in 2014 (p<0.001), and increased slightly in EDs from 0.22% in 2009 to 0.72% in 2017 (p = 0.0378) (Figure 1). In 2015, the estimate of HIV testing at visits to physician offices had a relative standard error that was too large to produce a reliable estimate. However, this point was included in the statistical analysis of the time trend for HIV testing at visits to physician offices. HIV testing occurred at a significantly higher percentage of preventive visits to physician offices and CHCs, compared with other visit types (Figure 2). HIV testing also occurred at a significantly higher percentage of visits where venipuncture was performed in physician offices, CHCs, and EDs, compared with visits without venipuncture. HIV testing among persons with private insurance increased at visits to CHCs from 1.55% during 2009–2012 to 2.67% during 2013–2014 (p = 0.0482); among those with Medicaid, testing increased at physician office visits from 0.39% during 2009–2012 to 0.84% during 2013–2014 and to 1.35% during 2015–2016 (p = 0.0352), and at CHC visits from 1.86% during 2009–2012 to 3.05% during 2013–2014 (p = 0.0287). HIV testing did not increase among persons with private insurance at physician office visits or among persons with either private insurance or Medicaid at ED visits.

**TABLE:** Mean number of annual visits by males and nonpregnant females aged 13–64 years to physician offices, community health centers, and emergency departments, and the percentage of those visits with a human immunodeficiency virus (HIV) test, by demographic and visit characteristics — United States, 2009–2017

Characteristic	Physician offices		Community health centers		Emergency departments	
	2009–2016		2009–2014		2009–2017	
	No. of visits*	HIV test, % (95% CI)	No. of visits*	HIV test, % (95% CI)	No. of visits*	HIV test, % (95% CI)
**Total**	**515,518,000**	**0.63 (0.45–0.87)**	**37,374,000**	**2.65 (2.29–3.07)**	**87,452,000**	**0.55 (0.45–0.66)**
**Sex**						
Female	305,086,000	0.62 (0.41–0.94)	24,349,000	2.56 (2.15–3.05)	48,378,000	0.54 (0.44–0.66)
Male	210,431,000	0.64 (0.49–0.84)	13,024,000	2.82 (2.40–3.33)	39,075,000	0.56 (0.45–0.69)
**Age group, yrs**						
13–19	48,606,000	0.56 (0.33–0.95)	4,029,000	2.45 (1.90–3.16)	10,695,000	0.53 (0.35–0.80)
20–29	57,179,000	1.71 (1.37–2.12)	5,764,000	5.08 (4.27–6.03)	21,311,000	0.62 (0.49–0.78)
30–39	77,948,000	1.02 (0.71–1.46)	6,725,000	3.65 (2.95–4.51)	17,751,000	0.60 (0.47–0.77)
40–49	110,264,000	0.67 (0.34–1.34)	7,864,000	2.26 (1.83–2.79)	16,371,000	0.53 (0.41–0.68)
50–64	221,520,000	0.21 (0.15–0.31)	12,992,000	1.36 (1.07–1.73)	21,324,000	0.45 (0.33–0.59)
**Race/Ethnicity**						
White	370,020,000	0.37 (0.30–0.45)	15,929,000	1.79 (1.49–2.13)	51,865,000	0.28 (0.22–0.36)
Black	57,345,000	1.51 (1.06–2.14)	6,116,000	4.30 (3.73–4.95)	20,888,000	1.07 (0.82–1.39)
Hispanic**^†^**	61,976,000	1.20 (0.70–2.04)	13,292,000	3.10 (2.39–4.00)	12,244,000	0.81 (0.62–1.07)
Other^§^	26,177,000	1.06 (0.42–2.65)	2,037,000	1.61 (0.99–2.61)	2,455,000	0.38 (0.20–0.72)
**U.S. region**						
Northeast	105,836,000	0.59 (0.43–0.81)	6,641,000	3.74 (3.00–4.66)	15,030,000	0.95 (0.64–1.40)
Midwest	102,923,000	0.38 (0.27–0.51)	6,266,000	2.00 (1.50–2.65)	20,583,000	0.49 (0.31–0.78)
South	192,637,000	0.80 (0.41–1.54)	8,900,000	2.91 (2.34–3.62)	33,848,000	0.53 (0.39–0.72)
West	114,122,000	0.61 (0.45–0.83)	15,567,000	2.31 (1.67–3.18)	17,992,000	0.30 (0.23–0.39)
**Metropolitan statistical area (MSA)^¶^**						
MSA	466,984,000	0.67 (0.47–0.94)	30,025,000	3.08 (2.63–3.59)	65,230,000	0.63 (0.51–0.78)
Non-MSA	48,534,000	0.28 (0.15–0.52)	7,348,000	0.93 (0.68–1.27)	12,724,000	0.11 (0.07–0.18)
**Insurance type**						
Private	347,585,000	0.61 (0.47–0.81)	6,612,000	2.15 (1.63–2.83)	29,199,000	0.42 (0.32–0.56)
Medicaid	51,315,000	0.79 (0.55–1.15)	14,591,000	2.95 (2.35–3.69)	24,027,000	0.67 (0.53–0.85)
Other**	90,670,000	0.32 (0.22–0.47)	13,626,000	2.63 (2.22–3.12)	26,594,000	0.48 (0.36–0.63)
**Provider specialty**						
Primary care**^††^**	263,192,000	1.09 (0.76–1.57)	18,599,000	2.47 (2.05–2.97)	—	—
Other	252,326,000	0.15 (0.11–0.22)	1,190,000	0.82 (0.32–2.06)	—	—
Nonphysician	—	—	17,585,000	2.97 (2.42–3.64)	—	—

**Abbreviation:** CI = confidence interval.

* Weighted nationally representative estimates.

**^†^** Hispanic/Latinos might be of any race.

^§^ Other races/ethnicities include Asian, Native Hawaiian or Other Pacific Islander, American Indian or Alaska Native.

^¶^ Location of health care venue.

** Other insurance types include Medicare, workers compensation, self-pay, no charge/charity, and other. Insurance type was missing for 6.0% of visits to physician office, 7.3% of visits to community health centers, and 8.2% of visits to emergency departments in the analytic sample.

**^††^** Primary care specialties include general and family practices, internal medicine, obstetrics and gynecology, and pediatrics.

**FIGURE 1:**
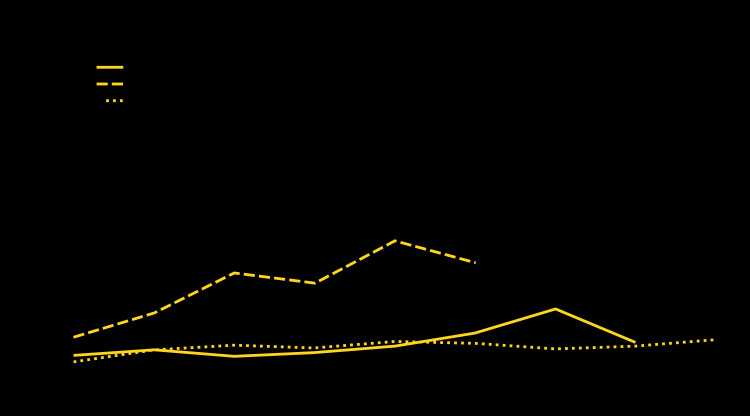
Human immunodeficiency virus (HIV) testing at visits made by males and nonpregnant females to physician offices,* community health centers, and emergency departments — United States, 2009–2017 *The estimate for HIV testing at visits made to physician offices in 2015 was not statistically stable. The trend for HIV testing in community health centers includes only physicians.

**FIGURE 2:**
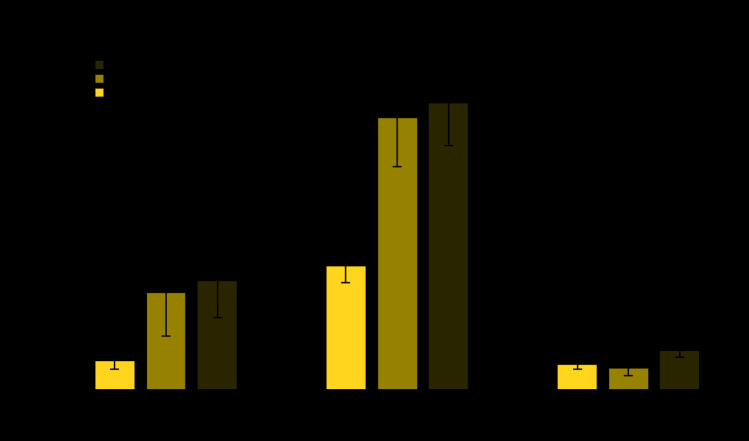
Human immunodeficiency virus (HIV) testing performed at visits made by males and nonpregnant females to physician offices, community health centers, and emergency departments, by type of visit* and whether venipuncture was performed at the visit — United States, 2009–2017 * HIV testing was estimated for preventive visits made to physician offices and community health centers, and for nonurgent care visits made to emergency departments. Percentages shown with 95% confidence intervals.

## Discussion

Although several hundred million visits were made annually to physician offices, CHCs, and EDs by persons aged 13–64 years during 2009–2017, HIV testing occurred at <1% of visits to physician offices and <3% of visits to CHCs. Overall, HIV testing increased in CHCs, a venue that serves populations with some of the highest rates of undiagnosed HIV infection. The higher percentage of visits that included HIV testing in CHCs might be attributed to the Health Resource and Services Administration’s (HRSA) efforts to increase HIV testing in primary care settings.**^,††^ HRSA has collected data on HIV testing since 1999 and has included these data as a quality measure in the Universal Data System reporting requirements for federally qualified health centers and look-alike health centers (community-based health centers that meet the requirements of the HRSA Health Center Program, but do not receive Health Center Program funding^§§^) since 2014.^¶¶^ Some CHCs have also implemented clinical decision support algorithms for increasing HIV testing (*5*,*6*).

HIV testing did not increase in physician offices during 2014–2016, despite elimination of patient cost-sharing, possibly because testing barriers unrelated to cost have not been addressed (e.g., dependence on busy providers to order HIV tests). In this study, an HIV test was performed more often at visits for preventive care. Preventive visits provide an ideal opportunity for HIV risk assessment to identify persons who require annual or more frequent testing and PrEP. An HIV test was also performed more often at visits with venipuncture, a convenient opportunity for including an HIV test when blood is drawn for other tests. Young black and Hispanic males and persons who inject drugs and who are at increased risk for acquisition of HIV might not have frequent preventive visits but do have health care visits for other reasons (*7*). Other types of visits can provide an opportunity for an HIV test, and these opportunities for testing persons in populations with the highest risk for acquiring HIV should not be missed. A modeling study estimated that a threefold increase in HIV testing rates at ambulatory care visits by black and Hispanic men aged 18–39 years would result in near-universal test coverage by age 39 years (*8*). HIV testing is easily performed with a simple blood test. Clinical decision supports can be developed that use information in EHRs to order an HIV test for patients who need one (*9*) and standing orders can increase routine opt-out testing (*10*), thereby reducing clinical barriers to HIV testing and increasing it at ambulatory care visits.

The findings in this report are subject to at least four limitations. First, this study cannot estimate the number of persons tested each year, because the sampling unit was a visit rather than a person; some persons might have had an HIV test at more than one visit. Second, smaller sample sizes of NAMCS and NHAMCS in recent years prevented analyses by patient and visit characteristics. Third, changes to the NAMCS and CHC sampling designs during the period of the study might have resulted in an underestimate or overestimate of HIV testing rates. Finally, recent data were not available, particularly CHC data that were only available through 2014; therefore, HIV testing in more recent years cannot be monitored for this important clinical venue.

Increasing HIV testing is a critical strategy for achieving the goals of the EHE initiative, and ambulatory health care encounters provide opportunities for increasing HIV testing that should not be missed. Jurisdictions participating in the first phase of the EHE initiative have the highest numbers of new HIV diagnoses and should be a focus of interventions to increase HIV testing. All persons should be routinely tested at least once during their lifetime and annually or more often if they are at increased risk for HIV infection because of sexual behavior or injection drug use, to identify those with HIV infection and link them to care, and to increase occasions for PrEP education and initiation. To end the HIV epidemic, testing of patients seeking care in ambulatory health care settings should be leveraged to increase the percentage of diagnosed infections and reduce HIV transmission.

**Summary**

**What is already known about this topic?**

CDC has recommended routine opt-out human immunodeficiency virus (HIV) testing since 2006, but the percentage of ambulatory care visits at which an HIV test is performed has remained low.

**What is added by this report?**

The percentage of visits with HIV testing increased in community health centers from 0.76% in 2009 to 2.41% in 2014 and in emergency departments from 0.22% in 2009 to 0.72% in 2017 but did not increase in physician offices during 2009–2016. HIV testing was performed at a higher percentage of visits for preventive care and visits with venipuncture.

**What are the implications for public health practice?**

To help end the HIV epidemic, health care systems can develop and implement clinical decision supports and training and accountability measures to increase HIV testing at ambulatory care visits especially in communities with high rates of HIV diagnoses.

## Footnotes

* https://www.uspreventiveservicestaskforce.org/Page/Document/UpdateSummaryFinal/human-immunodeficiency-virus-hiv-infection-screening.

† https://www.healthcare.gov/law/full.

§ https://www.cdc.gov/nchs/ahcd/index.htm.

¶ https://www.cdc.gov/nchs/ahcd/ahcd_estimation_procedures.htm.

** https://bphc.hrsa.gov/qualityimprovement/clinicalquality/hiv-aids/pal201013.html.

§§ https://www.hrsa.gov/opa/eligibility-and-registration/health-centers/fqhc-look-alikes/index.html.

¶¶ https://bphc.hrsa.gov/datareporting/reporting/index.html.
